# Traumatic Rupture of an Intermediate Tendon in a Patient with Patellar Duplication

**DOI:** 10.1155/2017/9475148

**Published:** 2017-01-31

**Authors:** Stéphane Pelet, Mathieu Hébert, Amerigo Balatri, Pierre-Alexandre LeBlanc

**Affiliations:** ^1^Centre de Recherche FRSQ du CHU de Québec, Hôpital de l'Enfant-Jésus, 1401 18ème rue, Ville de Québec, QC, Canada G1J 1Z4; ^2^Department of Orthopedic Surgery, CHU de Québec, Hôpital de l'Enfant-Jésus, 1401 18ème rue, Ville de Québec, QC, Canada G1J 1Z4

## Abstract

Patellar duplication is a rare asymptomatic condition. The diagnosis is often made following a traumatic event associated with an injury to the knee extensor mechanism. The treatment is often surgical and consists in removal of the smaller part of the patella with tendon reinsertion. The presence and rupture of an intermediate tendon between the two parts of the patella have not been reported in the modern literature. We present a traumatic rupture of an intermediate tendon in a patient with horizontal patellar duplication. The surgical management consisted of tenorrhaphy protected with a figure-of-eight tension band wire approximating the two parts of the patella. The patient recovered full knee range of motion and quadriceps strength at the last 8-month follow-up.

## 1. Introduction 

Patellar duplication is a rare asymptomatic condition. The diagnosis is often accidentally stated after a traumatic event leading to a weak knee extensor mechanism. Most cases are reported during adolescence and are consecutive to either a powerful quadriceps tensing or a fall [1]. The size of both parts can differ from one case to another and influences the surgical management in case of disruption.

The presence of an intermediate tendon connecting the two patellar parts was only described by Petty [2]. The ruptured intermediate tendon was excised and healing achieved through bone fusion of the two patellar parts. The knee biomechanics is directly related to the restoration of its initial anatomy, including the extensor mechanism length. An anatomical repair should provide a better functional outcome.

We present a traumatic rupture of an intermediate tendon in a 47-year-old patient with horizontal patellar duplication. An anatomical repair of the ruptured tendon was performed with an excellent functional outcome.

## 2. Case Report

A 47-year-old woman presented at our emergency room with anterior right knee pain, one day after a fall at home on level surface. She was unable to stand on her right leg. No previous trauma or knee pain was reported. Past medical history was positive for pituitary dwarfism and epilepsy. Physical examination revealed knee effusion and anterior hematoma. The knee was stable in all directions. Passive range of motion was complete, but the patient was unable to actively extend her knee. A complete extension lag was observed.

**Figure 1:**
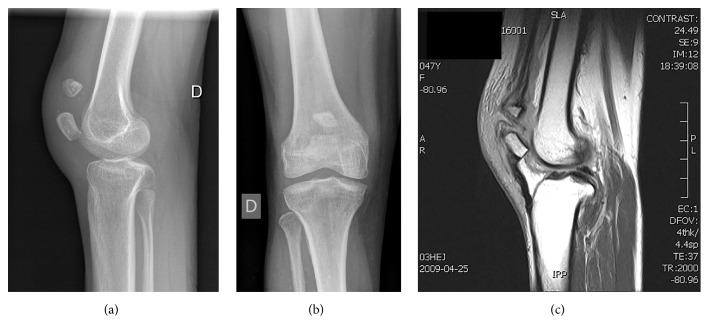
Lateral (a) and anteroposterior (b) knee views demonstrating a gap between the two parts of a duplicate patella. (c) Soft tissue is identified between the two parts of the patella (MRI T1 sagittal view).

Knee radiographs were obtained and demonstrated a significant diastasis between two patellar parts (Figures 1(a) and 1(b)). As both parts presented regular contours without evidence of acute fracture rims, further investigation was required. Magnetic resonance imaging (MRI) confirmed the presence of an intermediate ruptured tendon between the two patellar parts (Figure 1(c)).

The diagnosis of an intermediate tendon rupture in a duplicate patella was stated. In order to restore the integrity and length of the extensor mechanism, we proposed an anatomical repair of the intermediate tendon. The surgery was performed the same day.

### 2.1. Surgical Procedure

**Figure 2:**
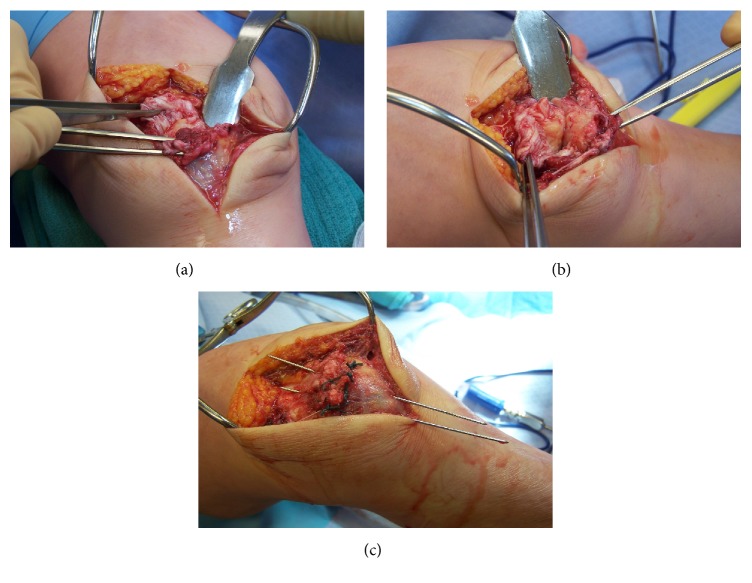
Surgical pictures presenting (a) the proximal and distal stumps of the ruptured intermediate tendon, (b) the use of two 1.6 mm K-wires to reduce both patella parts, and (c) the tenorrhaphy protected with two K-wires.

**Figure 3:**
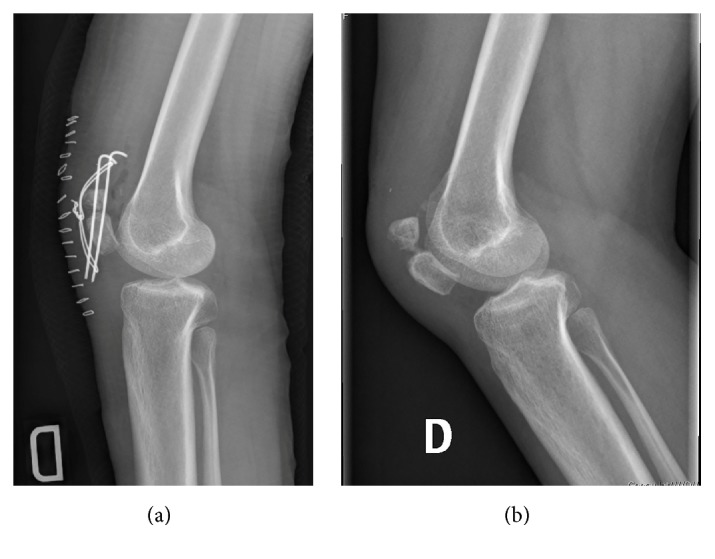
Immediate postoperative (a) and 8-month (b) lateral knee views.

Surgery was performed under general anesthesia, standard intravenous antibiotic prophylaxis, and tourniquet. A longitudinal 10 cm incision was centered on the patellar parts. Immediately after skin opening a voluminous hematoma was discharged at the level between the two patellar parts. A ruptured tendon was observed at this level. The two patellar parts were identified, without evidence of an acute or old fracture. A strip of tendon with the characteristics of a fully developed insertion was observed at both the distal part of the proximal patellar piece and the proximal aspect of the distal patellar part (Figure 2(a)). The medial and lateral retinacula were ruptured. The knee was positioned at 60° of flexion, and the two patellar parts were brought closer with two 1.6 mm K-wires without removing any tissue at the interface (Figure 2(b)). Tenorrhaphy was performed (without any excessive tension or shortening of the tendon) through multiple sutures with Ethibond 1 (Figure 2(c)). A figure-of-eight tension band wire was added to protect the tenorrhaphy (Figure 3(a)).

Postoperative care consisted of full weight-bearing with a hinged brace (blocked in full extension) for six weeks. Passive knee flexion was limited to 60 degrees during the first ten days and progressively increased to achieve 90 degrees after six weeks. Isometric quadriceps contraction was initiated immediately after surgery. Active knee motion (flexion and extension) and isokinetic quadriceps contraction were authorized after six weeks.

The tension band was removed after 6 months for slight discomfort, although the patient had already recovered a full active knee range of motion. The patient presented for a final visit 8 months after the surgery. She had a painless knee with full range of motion and symmetric quadriceps extension strength (no extension lag). The last radiographs illustrate a typically healed duplicate patella with a free space between the two parts, as bone healing was not the goal (Figure 3(b)).

## 3. Discussion

Two etiological theories explain the development of a duplicate patella. The first hypothesis refers to a congenital malformation. This assertion is supported by the report of bilateral horizontal cases [1] and the observation of coronal [3] and frontal patellar duplication (also called double layer patella, reported in patients with multiple epiphyseal dysplasia) [4]. A fully developed tendon between the two patellar parts was described in these reports.

The second hypothesis considers the duplicate patella as the result of the natural healing of an undiagnosed sleeve fracture. This fracture occurs in skeletally immature patients and represents a traumatic avulsion of the extensor mechanism with a small sleeve of cartilage and periosteum. These fractures can occur at either the proximal or the distal pole of the patella. The treatment is mainly surgical; however, some cases were successfully treated with braces [1]. The osteogenic potential of the cartilage sleeve explains the evolution towards a duplicate patella [5, 6].

The present case occurred in a patient with pituitary dwarfism. The two patellar parts have a significant size and the patient did not report any previous knee trauma. A previous sleeve fracture is unlikely. A duplicate patella is not commonly related to patients with dwarfism from any cause, and we were unable to find other reported cases in the literature with this kind of association. The duplicate patella's etiology remains unclear.

Most patellar duplications are asymptomatic [7] and surgery is reserved for cases when the continuity of the knee extensor mechanism is broken. The goal of the surgical management is to achieve good healing of the extensor mechanism with restoration of its initial length. Excessive shortening will result in a loss of knee flexion and lengthening in an extension lag.

The main surgical options are excision of the small fragment and tendon reinsertion into the remaining patellar part, rigid fixation of both parts in order to achieve bone healing, or anatomical repair of the intermediate tendon.

This case report is the first description of an intermediate tendon rupture in a patient with horizontal patellar duplication, treated with an anatomical repair. The treatment's choice was mainly influenced by the size of both fragments. An excision of the proximal patellar part and the resection of the intermediate tendon in order to achieve bone fusion would both result in a significant loss of flexion (we estimated in this case 2 to 3 cm extensor mechanism shortening with these options). An anatomical repair was temporarily protected by a tension band. The tendon suture was performed in 60-degree flexion in order to prevent overtightening. The original length of the extensor mechanism was restored, and the final outcome was excellent with a full knee range of motion and symmetrical complete quadriceps strength. The remaining gap between the two patellar parts on the final lateral knee view, with an anatomical patellar height, illustrates the characteristics of an intact horizontal duplicate patella with an intermediate tendon.

Petty reported a similar case in 1925, with rupture of an intermediate tendon in a horizontal duplicate patella [2]. The treatment consisted in bone fusion. However, the report did not describe the nature of the intermediate tendon. The final clinical outcome is unknown except that bone fusion was achieved.

Cipolla et al. reported six patients with rupture of the knee extensor mechanism and duplicate patella [8]. The treatment consisted in excision of the smaller patellar part and tendon reinsertion. The final clinical outcome was considered good with a 5-degree loss of knee flexion and some extension lag. All cases consisted of very small patellar parts (or even calcifications). The diagnostic etiology of a previous sleeve fracture was proposed. This small series differed from this case report as no cases were significant horizontal double patella. The presence and nature of the intermediate tendon were also not reported.

Patellar duplications should not be confused with bipartite patella. The development of bipartite patella is related to an anomaly in the ossification process. An original classification was proposed by Saupe [9]; type I is apical, type II lateral, and type III superolateral (the most frequent). Type I was excluded from bipartite patella by many authors since the presence of an apical ossification nucleus has never been demonstrated [10]. This type probably corresponds to a double patella. So-called bipartite patella fractures correspond to an injury to the intermediate tissue between the two patellar parts and have usually no influence on the knee extensor mechanism. Nonsurgical management is proposed for most cases [11] and surgery reserved for the rare cases with an injury of the extensor mechanism [12–16].

The need for complementary imaging is mandatory to diagnose the extensor mechanism rupture and identify an intermediate tendon in a patient with a painful double patella. We propose to request knee MRI in every patient presenting with a complete extension lag and a lateral knee radiograph describing a gap between two patellar parts with suspicious fracture rims.

This case illustrates the importance of a precise initial diagnosis. We should think of a double patella with intermediate tendon rupture in all cases when the two separated patellar fragments are atypical. Differential diagnosis with a bipartite patella is necessary. Complementary imaging helps to best understand the lesion. An anatomical tendon repair can provide an excellent clinical outcome.
